# Spinal Muscular Atrophy Diagnosed by Newborn Screening

**DOI:** 10.15844/pedneurbriefs-33-5

**Published:** 2019-12-31

**Authors:** Matias Lopez-Chacon, Amber N. Buehner, Vamshi K. Rao

**Affiliations:** 1 Division of Neurology, Ann & Robert H. Lurie Children’s Hospital of Chicago, Chicago, IL; 2 Department of Pediatrics and Neurology, Northwestern University Feinberg School of Medicine, Chicago, IL

**Keywords:** Spinal Muscular Atrophy, Newborn Screening, Guidelines

## Abstract

A panel of experts representing academic centers, family foundations and pharmaceutical industry came together to formulate a treatment algorithm for infants diagnosed via newborn screening (NBS) with Spinal muscular atrophy (SMA).

A panel of experts representing academic centers, family foundations and pharmaceutical industry came together to formulate a treatment algorithm for infants diagnosed via newborn screening (NBS) with Spinal muscular atrophy (SMA). The premise was based on the fact that 95% of SMA is due to a homozygous deletion of SMN1 and that disease severity is ameliorated by number of copies a paralog, SMN2. Treatment guidelines, after positive identification of SMA, could therefore be centered on the SMN2 copy number. The panel of 15 experts reached consensus using a modified Delphi process.

The panel was unanimous in their recommendations for infants with 2 or 3 copies of SMN2 (predicted SMA type 1 or 2) to receive immediate ‘SMN up regulating’ therapies. For, symptomatic neonates and 1 copy of SMN2 (predicted SMA type 0), the consensus was to defer to the attending physician for assessment of treatment benefit whereas for the truly pre-symptomatic, immediate treatment was strongly recommended.

The panel was divided for patients with 4 SMN2 copies (predicted SMA type 3 or 4), but did reach consensus advocating for no immediate treatment and careful follow up for presentation of symptoms. Follow up was to be with a neuromuscular specialist and a place that could identify an exact SMN2 copy number and disease modifying mutations. The visits were to happen every 3 to 6 months until age 2 years and every 6 to 12 months thereafter. During such visits appropriate tests, such as electromyography (EMG), compound muscle action potentials (CMAP), myometry, physical examination (PE), and motor function scales (MFS) were recommended. Active or chronic changes on EMG, results below normative values for CMAP, clinically meaningful reductions in myometry for age, changes in PE (such as loss of reflexes, failure to meet milestones or motor regression, proximal or trunk righting/derotational weakness) and failure to gain/loss of motor milestones based on MFS, would prompt initiation of treatment. In addition, a written checklist was recommended for caregivers whose children were diagnosed with SMA on NBS, as their course would likely be different than those identified after symptom onset. The presence of key signs on the checklist, would in turn prompt an immediate re-evaluation. [1]

COMMENTARY. There is evidence of irreplaceable loss of motor neurons and severe denervation in the first 3 months of life with more than 90% of motor unit loss by 6 months in type 1 SMA. Although, mean age of onset of symptoms was 2.5, 8.3 and 39 months, there is mean delay in genetic diagnosis of 3.6, 14.3 and 43.6 months for types 1, 2, and 3 respectively [2].

Earlier institution of treatment has been proven, in clinical trials with both SMN2 upregulating and gene replacement therapy for patients with 2 copies of SMN2, to show higher motor-milestone response and survival than the control group [3,4]. The current study, based on NBS and SMN2 copies, provides a useful template to the child neurologist for instituting both time sensitive treatments and appropriate follow up for children with SMA in the future. However, one must keep in mind that although certain follow up guidelines have been laid out for individuals with 4 or more copies of SMN2, both emerging NBS data and differing treatment strategies for SMA will additionally distill these guidelines over time.

## Disclosures

The authors have declared that no competing interests exist.
